# Update of: Kara et al., The BetterBirth Program: Pursuing Effective Adoption and Sustained Use of the WHO Safe Childbirth Checklist Through Coaching-Based Implementation in Uttar Pradesh, India

**DOI:** 10.9745/GHSP-D-18-00065

**Published:** 2018-03-21

**Authors:** Nabihah Kara, Rebecca Firestone, Tapan Kalita, Atul A Gawande, Vishwajeet Kumar, Bhala Kodkany, Rajiv Saurastri, Vinay Pratap Singh, Pinki Maji, Ami Karlage, Lisa R Hirschhorn, Katherine EA Semrau

**Affiliations:** a Ariadne Labs, a Joint Center between Brigham and Women's Hospital and the Harvard T.H. Chan School of Public Health, Boston, MA, USA.; b Population Services International, Washington, DC, USA.; c Population Services International, Lucknow, Uttar Pradesh, India.; d Ariadne Labs, Boston, MA, USA; Department of Health Policy and Management, Harvard T.H. Chan School of Public Health, Boston, MA, USA; Department of Surgery, Brigham and Women's Hospital, Boston, MA, USA.; e Community Empowerment Lab, Lucknow, Uttar Pradesh, India.; f Jawaharlal Nehru Medical College, Belgaum, Karnataka, India.; g Ariadne Labs, Boston, MA, USA; Feinberg School of Medicine, Northwestern University, Chicago, IL, USA.; h Ariadne Labs, Boston, MA, USA; Department of Medicine, Harvard Medical School, Boston, MA, USA; Division of Global Health Equity, Department of Medicine, Brigham and Women's Hospital, Boston, MA, USA.

See updated article.

In the article “The BetterBirth Program: Pursuing Effective Adoption and Sustained Use of the WHO Safe Childbirth Checklist Through Coaching-Based Implementation in Uttar Pradesh, India” by Nabihah Kara, Rebecca Firestone, Tapan Kalita, et al., which appeared in the June 2017 issue (Volume 5, Number 2) of GHSP, the authors describe the BetterBirth program and lessons learned as the program was tested in a cluster-randomized controlled trial conducted in Uttar Pradesh, India. The trial assessed whether use of a safe childbirth checklist could improve birth attendants' adherence to essential birth practices and maternal and newborn health outcomes. At the time of publication of this article, we noted at the end of the Abstract field that we would update this article by including a reference to the impact findings of the intervention, which were pending publication in another journal. Those impact findings have now been published in the *New England Journal of Medicine*, so we have updated the related GHSP article accordingly. The overall impact findings were that the intervention had no significant effect on maternal or perinatal mortality or maternal morbidity, despite having positive effects on essential birth practices.

These impact findings are now referenced at the end of the Abstract.

